# Radiographic Evaluation of Children with Febrile Urinary Tract Infection: Bottom-Up, Top-Down, or None of the Above?

**DOI:** 10.1155/2012/716739

**Published:** 2011-08-11

**Authors:** Michaella M. Prasad, Earl Y. Cheng

**Affiliations:** Division of Urology, Children's Memorial Hospital, 2300 Children's Plaza, P.O. Box 24, Chicago, IL 60614, USA

## Abstract

The proper algorithm for the radiographic evaluation of children with febrile urinary tract infection (FUTI) is hotly debated. Three studies are commonly administered: renal-bladder ultrasound (RUS), voiding cystourethrogram (VCUG), and dimercapto-succinic acid (DMSA) scan. However, the order in which these tests are obtained depends on the methodology followed: bottom-up or top-down. Each strategy carries advantages and disadvantages, and some groups now advocate even less of a workup (none of the above) due to the current controversies about treatment when abnormalities are diagnosed. New technology is available and still under investigation, but it may help to clarify the interplay between vesicoureteral reflux, renal scarring, and dysfunctional elimination in the future.

## 1. Introduction

Three studies are commonly employed in the workup of febrile urinary tract infections (FUTIs): renal-bladder ultrasound (RUS), voiding cystourethrogram (VCUG), and dimercaptosuccinic acid (DMSA) scan. This paper will discuss the rationale behind the timing of these studies (“top-down” versus “bottom-up” methodology), along with the individual advantages and limitations of each approach. The debate involves the ideal outcome of interest vesicoureteral reflux (bottom-up) or renal parenchymal involvement (top-down). The controversy on this topic has swelled to the point that certain forums are even promoting a more limited workup (none of the above) for a subset of patients. Finally, new techniques and technologies using magnetic resonance (MR) urography and voiding urosonography (VUS) are emerging. These innovative studies may impact management strategies in the future. The purpose of this paper is to assess the current literature on bottom-up and top-down approaches as well as newer modalities and to evaluate the association between vesicoureteral reflux and renal scarring as it pertains to the workup of a child with FUTI.

## 2. Background

Urinary tract infections (UTIs) in young children are common with an overall prevalence of 7.0% among infants presenting with fever and a pooled prevalence of 7.8% among children with urinary symptoms [1]. This diagnosis often leads to a radiographic workup to look for correctable urinary tract abnormalities that may predispose the child to infection. The objective is to identify which patients are susceptible to renal damage. Ideally, medical or surgical interventions can then be employed to prevent this cohort from developing future infections or sustaining further injury (although this point is also controversial). Scarring from repeated infections of the renal parenchyma leads to hypertension in 10 to 20% of patients [2]. Also, reflux nephropathy progresses to end-stage renal disease requiring dialysis or transplantation in 10 to 25% of patients worldwide [2–5]. Thus, the radiographic workup is critical in determining appropriate therapy, but there is little agreement as to whether the emphasis should be on the appearance of the kidneys versus the presence of VUR. 

**Figure 1:**
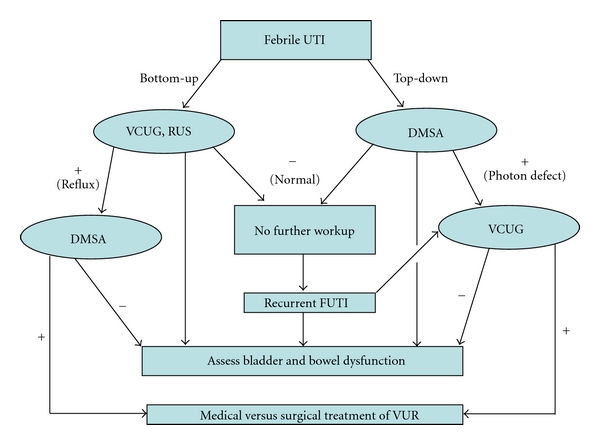
A schematic of the bottom-up versus top-down approach.

Historically, the studies most commonly used for this purpose were a RUS and VCUG. Collectively, this is now referred to as the “bottom-up” approach. This method relies on renal-bladder ultrasound (RUS) to identify anatomic irregularities (renal parenchymal defects) or evidence of obstruction. The voiding cystourethrogram (VCUG) targets lower urinary tract abnormalities and detects vesicoureteral reflux (VUR). Patients diagnosed with reflux or parenchymal deformity may undergo a DMSA scan at a later date to assess for scarring (Figure 1). Alternatively, the “top-down” approach targets the kidney at the outset with a DMSA scan to diagnose acute renal parenchymal involvement at the time of the FUTI. Patients that have photon defects or evidence of parenchymal inflammation are subsequently referred for a VCUG to assess for reflux in addition to a late DMSA (6–12 months) to assess for permanent scarring [6]. 

Although it seems to be a minor point, the critical issue is the order in which these tests are performed. With either method, a negative study will obviate the need for further investigation. For the bottom-up approach, fewer DMSA scans will be performed. The same will be true for VCUG studies in the top-down approach. Why do two distinct methodologies exist? In brief, the VCUG is a stressful study for the patient and family due to the need for catheterization [7, 8]. In addition, it identifies a population with VUR that may never be clinically significant. Thus, the top-down approach has been advocated in some circles because a negative DMSA precludes the need for a catheter and a positive study identifies the cohort that is most “at-risk” for subsequent renal scarring. However, each approach has merits and flaws; these points will be reviewed below. 

## 3. Radiographic Options

### 3.1. Bottom-Up Approach

Voiding cystourethrogram (VCUG) is the gold-standard exam to assess for vesicoureteral reflux (VUR). However, only 30–40% of children with a UTI will have reflux, which suggests that over 60% of VCUG tests ordered for this indication may be unnecessary [9, 10]. The 1999 American Academy of Pediatrics (AAP) practice guidelines recommend the regimen we now recognize as the “bottom-up” approach to FUTI based on the association between UTIs and urinary tract abnormalities [11]. At the time, it was thought that VUR was necessary for renal scarring to occur, so the presence of reflux was used a surrogate for the true endpoint of interest: renal parenchymal injury. 

Now it is recognized that the actual relationship between VUR and renal scarring is poorly understood. In other words, one does not have to have reflux to develop a scar, and one does not always develop scarring with a reflux-associated UTI. However, numerous studies continue to support an association between reflux and scarring. A recent prospective study of 227 children hospitalized with their first FUTI found that VUR continues to be a significant predictive factor for acute pyelonephritis (APN) featuring renal parenchymal involvement and ultimate injury [12]. More patients with VUR presented with acute photon defects on initial DMSA scan (*P* = 0.034) and progressed to scar formation (*P* = 0.004). Surprisingly, even though there was an association between VUR grade and acute photon defects (*P* = 0.001), there was no correlation between VUR grade and subsequent scar formation (*P* = 0.279). A recent systematic review of the published literature revealed that children with VUR were significantly more likely to have APN and renal scarring versus those without VUR (RR 1.5 and 2.6, resp.) [13]. In this paper, scarring was more likely with grade III or greater VUR (RR 2.1). Furthermore, there are higher rates of febrile infections in children with VUR and UTI [9]. In summary, the relationship between VUR and renal scarring is clearly not 1 : 1; however, some association undeniably exists, and this forms the basis for the VCUG recommendation to identify those individuals with reflux that may be at subsequent risk for secondary scarring. 

The bottom-up tactic will readily identify reflux, but it is difficult to predict which subset of patients will spontaneously resolve and which will have harmful sequela of their disease. Thus, a proportion of patients with reflux will undergo the morbidity of surveillance and potentially be subjected to overtreatment. A recent prospective observational study followed 115 infants diagnosed with high grade (III to V) VUR [14]. Spontaneous complete resolution of VUR was reported in 30 patients (26%). Another 14 (12%) were downgraded to grade I to II reflux for an overall resolution rate of 38%. The median age at which resolution occurred was 27 months [14]. As mentioned above, VUR does not always lead to scarring in the setting of a FUTI [2, 15, 16]. Similarly, although VUR has been associated with renal injury, there is a 30% chance in children <1 year and 37% chance in those aged 1–5 years that scarring may exist in the absence of this abnormality [16, 17]. These findings indicate that the VCUG will only partially identify the population that is at-risk (because VUR may spontaneously resolve and not all VUR + UTI = scar) and it will miss a subset of children that experience renal scarring without VUR.

The other component of the bottom-up approach is RUS; although, it would not be uncommon to see this study ordered in conjunction with the top-down approach as well. Ultrasound is the most common initial intervention for a child with febrile or afebrile UTI. It provides a gross anatomic assessment of the urinary tract that is noninvasive and does not use ionizing radiation. However, given the current frequency of prenatal ultrasounds, most children with congenital obstruction of the urinary tract are diagnosed before birth and treated before the first UTI occurs [8, 18]. This suggests that an infant with uncomplicated FUTI does not need a RUS if a reliable assessment was performed in the third trimester. Ultrasound is an integral part of most urologic workups and surveillance such that it is often a knee-jerk reaction to order one upon referral; however, the added benefit that this study contributes for an infant with FUTI is negligible in the era of prenatal screening. 

Several studies have found that ultrasound fails to alter management over DMSA scanning or VCUG [18]. Mahant et al. [19] observed that RUS only carries a sensitivity and specificity of 40% and 76%, respectively, for VUR. Certainly the VCUG and DMSA scan in combination are capable of diagnosing a wide range of genitourinary anomalies including VUR, posterior urethral valves, duplex systems, moderate to severe hydronephrosis, and anatomic or function renal asymmetry [20]. Although it is widely available, the quality of the study is technician dependent, it does not provide a quantitative assessment of renal function, and it is not sensitive enough to detect all scarring. On the other hand, it is often comforting to the family to have a safe, noninvasive exam that grossly reveals the condition of the kidneys. While there may not be sufficient evidence to say that RUS is absolutely necessary to the workup, it is very reasonable to include it.

### 3.2. Top-Down Approach

DMSA has replaced the intravenous urogram as the gold-standard study to assess for acute renal inflammation and established parenchymal injury (scarring). One basic tenet of the top-down approach is that an early DMSA renal scan (in the acute phase of the febrile illness) can detect renal involvement that signifies the kidney is vulnerable to subsequent injury. Thus, one only obtains a VCUG in those patients that have evidence of a parenchymal defect on a DMSA renal scan. This will theoretically identify a more vulnerable population with reflux, one that has parenchymal involvement with a FUTI, and lessen the need for VCUGs in all patients with FUTI. This viewpoint recognizes renal parenchymal infection, rather than VUR, as the nidus for acquired scarring [15, 21, 22]. Serial DMSA scans show that if new scars develop, they are localized to previous sites of inflammation. 

It bears mentioning that there is a lack of consensus about the etiology and significance of scars seen on initial DMSA or RUS. It has been argued that congenital dysplasia may account for these defects rather than an acute insult to the renal parenchyma from infection and/or reflux because infants with antenatal hydronephrosis that never experienced a UTI can have an abnormal DMSA study [21, 23]. Part of this argument considers scarring and VUR to be a global development problem where the entire unit from kidney to ureter is abnormal through embryologic development as opposed to an evolving, acquired event after birth [23, 24]. As with the bottom-up approach, the concern is that an abnormal DMSA scan will mistakenly identify children with congenital dysplasia as part of the population susceptible to further injury when, in fact, their lesions are static. Nevertheless, acute pyelonephritis (APN) leads to detectable inflammation on acute DMSA imaging and will cause subsequent scarring in at least 50% of patients [24, 25]. In either scenario, a subset of children will potentially be subjected to over-treatment. However, the morbidity of surveillance in a child with clinically insignificant reflux is arguably greater than the conservative management of a child with a congenitally dysplastic kidney.

A common criticism of the top-down approach is that it will miss VUR that does not have renal involvement. However, the majority of missed cases tend to be low-grade or grade III VUR that resolves or improves on followup and is not clinically significant (defined as dilating grade III-IV VUR, or VUR associated with recurrent UTI, scarring, or significant dysfunctional elimination) [2, 21]. A recent prospective trial of 121 children presenting with a first FUTI showed APN on 88 (73%) of acute phase (less than 7 days after fever or symptom onset) DMSA studies [2]. VUR was present in 78 patients (64%). The abnormal acute DMSA predicted clinically significant VUR with an odds ratio of 35.4. These children were followed for 5 years with repeat DMSA performed at 6 months. 26 (21.5%) of the 88 initially abnormal studies showed subsequent renal scarring. Of this latter group, 14 had VUR and 12 did not. The initial DMSA detected 95% of the patients with clinically significant VUR, and the top-down approach would have avoided 42 (35%) VCUGs in patients who did not develop scarring [2]. Moorthy et al. [26] reviewed the records of 108 children presenting with a first UTI under the age of 1 year. Of 216 renal units (RU) that were normal on RUS, 8 had scarring on DMSA 3–6 months after presentation. Although VUR was demonstrated in 25 RU, only 4 had concomitant scarring. In summary, these studies reinforce the notion that renal involvement and scarring can exist in the presence and absence of VUR. Thus, the VCUG can safely be an adjunct study to the principal DMSA exam to better identify an at risk cohort. The DMSA defines the susceptible population, and the VCUG identifies the proportion that may be at risk for further parenchymal damage and should be considered for antibiotic prophylaxis and/or surgical intervention.

The acute phase DMSA is a critical component of the top-down approach as late phase exams may miss children who present with repeat FUTI that does not result in scarring [15, 24]. In the previously cited study, the sensitivity and specificity for acute phase DMSA to detect clinically significant VUR were 95.7% and 71.9%, respectively [2]. Late phase DMSA scans had a sensitivity and specificity of 27.5% and 76.9%, respectively, for the same population. The natural history of DMSA defects was studied prospectively to assess the impact of scars on renal growth [26]. 50 children with scarring on DMSA scan 6 months after an episode of APN demonstrated improvement in 72% of lesions over a 3-year period, regardless of VUR status. Although a higher number of scars was associated with higher grades of VUR (*P* = 0.02), the number of scars observed at 6 months, and not the severity of VUR, was associated with impaired renal growth (*P* < 0.001, *P* = 0.34, resp.). Although some kidneys are vulnerable to injury, this study suggests that not all defects seen at 6 months are clinically significant because they improve over time [26]. In summary, the appearance of the kidneys on DMSA can evolve. Thus, obtaining a study during the initial episode of FUTI is critical to identifying the population with kidneys susceptible to injury and repeat febrile infection, even though this may not result in a definitive scar. 

Exposure to ionizing radiation remains as a common criticism for both VCUG and DMSA studies. It has been argued that, historically, VCUG transmits a larger dose of radiation to the pediatric gonads in comparison to DMSA [20]. However, DMSA carries a 10-fold higher radiation dose than pulsed fluoroscopy, which is currently used in most centers for VCUG exams [27]. Nevertheless, the radiation during fluoroscopy is focused on the pelvis while with a DMSA study, it is diffused throughout the body with concentration in the kidneys. Sequential exams for surveillance would increase this exposure. Additional limitations to the DMSA scan include the need for intravenous access and sedation, lack of availability at all centers, inconsistent interpretation of the exam, and the longer duration of the test (1-2 hours) as compared with VCUG. On the other hand, the VCUG requires urethral catheterization which can many times be a focus of discomfort and anxiety for both the child and parents. In addition, a significant proportion of children will undergo surveillance for a disease that may never be clinically significant. Thus, the search continues for the optimal methodology of evaluation as well as for alternative imaging techniques that can overcome the risks and inadequacies mentioned (Table 1).

### 3.3. Emerging Technology

As the diagnostic paradigm for FUTI is evolving, so is the technology available to practitioners. The bottom-up versus top-down debate may be altered significantly by the radiographic options on the horizon as they change diagnostic capabilities and the understanding of disease processes. Magnetic resonance urography (MRU) can provide both anatomic and functional data in one study. Due to the improved spatial and contrast resolution, congenital renal dysplasia can be differentiated from acquired renal damage on MRU [28]. Chang et al. [29] showed a direct relationship between the renal parenchymal damage and volume detected by MRU and VUR grade. In a retrospective review of 114 patients with reflux, and 21 nonrefluxing controls, MRU was able to detect a renal size discrepancy between the two groups. This size discrepancy persisted in the comparison of contralateral nonrefluxing kidneys with nonrefluxing controls and occurred in the absence of focal scarring (*P* < 0.0001). This data supports the notion that patients with VUR can have abnormal embryological development or hypoplasia before birth and the first insult of a UTI. Alternatively, the contralateral nonrefluxing kidney may be impacted by bilateral pyelonephritis initiated by the refluxing kidney. The etiology is still unclear, but the association between VUR and APN could be characterized more completely if MRU assessment is included in future studies. 

Another magnetic resonance imaging technique has been developed to perform interactive voiding cystourethrography (iMRVC), which involves using a pulse sequence and rapid switching between views to permit prolonged dynamic imaging of the urinary tract [30]. A feasibility study in unsedated infants was performed on 12 patients with a first UTI or abnormalities on early postnatal US [30]. VUR was identified in 5 children using iMRVC versus 3 using conventional VCUG (sensitivity 100%, specificity 83% for iMRVC). It should be noted that the iMRVC studies followed a single cycle VCUG study, and subsequent bladder filling cycles have been known to increase VUR detection. The iMRVC studies obtained adequate images of the urethra, and the technique was refined over the course of the trial such that the imaging time dropped from 60 minutes to 20 minutes, commensurate with the time required for a VCUG [30]. 

Both MRU and iMRVC show promise for future use. They offer exquisite anatomic detail in conjunction with dynamic, functional information without the need for radiation. These tests are expensive to administer, require sophisticated processing techniques, and may require sedation in younger patient populations. Also, intravenous gadolinium in MRU studies needs to be used with caution in patients with severe renal impairment due to the risk of a rare but disabling complication known as nephrogenic systemic fibrosis [28]. For research purposes, these modalities can provide more information about the relationship between VUR, APN and subsequent renal scarring than current methodologies. However, these tests do not yet shed light on the optimal approach to a patient with FUTI. In terms of clinical practice, the demand for this imaging modality may outpace the research to support it in this era characterized by the rapid adoption of new technology.

To come full circle, the ultrasound modality may be experiencing a resurgence at certain centers. Voiding urosonography (VUS) has been refined over the last ten years with the addition of harmonic imaging and second-generation contrast agents to improve accuracy. A recent comparison of VUS with VCUG studied 183 children (366 RU) with UTI, upper tract dilation, or previously diagnosed VUR [31]. VUR was detected in 140 RU based on the presence of contrast material in the ureter or pelvicalyceal system during the exam; however, VCUG only picked up 14 cases whereas 37 cases were identified with VUS. This may be the result of continuous imaging using sonography versus intermittent fluoroscopy. The prevailing criticism of this technique is that the urethra cannot be properly visualized. However, Duran et al. [32] showed that recent technological advances have enabled diagnosis of posterior urethral valves and diverticula of the prostatic utricle and the anterior urethra in boys. The bladder neck and urethra were felt to be adequately visualized in 150 patients included in the study. This is consistent with prior reports using this technique [33]. Like iMRVC, this exam still requires catheterization. Unlike iMRVC and MRU, VUS is less costly and may be more accessible to the general population. However, the techniques are still operator-dependent and require highly skilled sonographers.

### 3.4. Other Considerations

Risk factors for scarring have traditionally included increasing grade of VUR, increasing frequency of UTI, and presence of bladder and bowel dysfunction, otherwise known as dysfunctional elimination [9]. The irony of the top-down approach is that it brings renewed focus to this type lower urinary tract dysfunction. Children with APN and no evidence of VUR are often found to have dysfunctional elimination (DE) [24]. An aggressive, detailed assessment and treatment of bladder and bowel dysfunction may identify ways to decrease recurrent UTI [9, 34]. High bladder capacity and increased residual urine are used as markers of bladder dysfunction. These variables, along with renal abnormality on DMSA and recurrent FUTI, were negative predictors of VUR resolution in a prospective observational study of 115 children with reflux [14]. In the absence of these three factors, there was a 91% probably of spontaneous resolution by the age of 3 years in children with grade III–V VUR. Furthermore, the propensity for scarring is diminished when DE is effectively treated [2]. DE is a well-established risk factor for recurrent UTI, persistent VUR, failed medical and surgical interventions, and acquired scarring [22, 34]. The merits of medical or surgical treatment have been hotly debated in the literature; however, the critical intervention for everyone may be behavioral therapy to address the abnormal bladder and bowel function (Figure 1). Thus, regardless of what type of radiographic evaluation is done for a child with a FUTI, the more clinically important intervention may be to identify those children with FUTI that have dysfunctional elimination and treat it.

What may be missing from all of the discussion regarding the best type of radiographic evaluation to address children with FUTIs that are at-risk for renal scarring is the fact that FUTI (independent of scarring) remains a source of considerable illness and morbidity. Hospitalizations for children with FUTI cost an estimated $180 million per year in the United States [24]. Although the surgical correction of VUR has not been shown to reduce the incidence of infections or scarring, it is more effective than prophylactic antibiotics in preventing episodes of febrile infection [35]. The timing and duration of prophylactic antibiotics in the setting of FUTI have been controversial, but evidence is accumulating in favor of their use. The Swedish Reflux Trial showed that female patients on prophylaxis may continue to have FUTI but are less likely to have new renal scars [23]. A formal Cochrane Database review published in 2011 considered all randomized controlled trials (RCT) and quasi-RCTs that assessed antibiotic prophylaxis versus placebo or no treatment to prevent recurrence of UTI in children [36]. The authors concluded that a small but consistent benefit exists in favor of low-dose antibiotic prophylaxis for reducing repeat infections. 

Recent evidence suggests that it is not the severity of illness that puts kidneys at risk for deterioration but rather a delay in appropriate therapy [4, 12]. According to both clinical evidence and basic science research using mature sows, there is no age limit to developing renal scarring, contrary to previous theories [4, 5, 37]. Thus, prompt treatment of infections remains relevant as long as reflux persists. The purpose of identifying reflux or scarring in this cohort is twofold: first, to maintain a heightened awareness that these patients in particular require timely diagnosis and management of a febrile illness; second, to institute behavioral strategies for more effective bladder and bowel elimination. These basic interventions are less likely to be recommended if the patient is never referred to a specialist for formal evaluation. To be sure, each imaging technique has its flaws, but until properly controlled studies are available to guide our diagnostic efforts, the status quo should be maintained not abandoned.

To add to the confusion, guidelines from the National Institute for Health and Clinical Excellence (NICE) in the United Kingdom suggest that routine imaging for VUR following a typical, uncomplicated UTI is not required [38]. Infants with atypical UTI (defined by severity of illness, poor response to antibiotics, or a non-*E. coli* infection) or a recurrent UTI are the only ones recommended to undergo a limited workup. This tapered approach represents a dramatic deviation from previous standards and reflects the opinion that congenital dysplasia may represent a benign etiology [37, 38]. In addition, the high spontaneous resolution rates for some forms of uncomplicated VUR are often cited in favor of a more limited approach [14]. Also, there has been renewed emphasis lately on reducing ionizing radiation in the pediatric population, which argues against an aggressive or indiscriminate workup of FUTI [39]. However, a recent meta-analysis showed no reduction in the proportion of children with reflux nephropathy progressing to ESRD despite our current intervention strategies [28, 40]. That statistic will not improve with the emerging trend to reduce the evaluation of patients with FUTI. Certainly it would be better to focus our efforts on patients that are most at-risk for renal deterioration, but that population has not been satisfactorily defined at this time.

Although renal parenchymal injury is the most severe long-term sequela of FUTIs, the morbidity and costs of recurrent urinary tract infections (especially febrile UTI) should not be underestimated. Thus, the potential for renal scarring is one target to identify, but the prevention of FUTI must be included in the decision-tree algorithm. As we have seen with the NICE guidelines, some nonurologic forums have abandoned all consideration of top-down or bottom-up methodologies; they are beginning to adopt “none of the above” strategies instead. While the evidence is clearly not in favor of one approach over the other, the outcomes are potentially damaging enough to warrant some radiographic investigation, surveillance, and management by a specialist. Although the diagnosis of reflux or scarring carries an unknown long-term predictive value, it heightens awareness among practitioners and the family such that the child is properly monitored and treated for further febrile events.

## 4. Conclusions

Currently, the only noncontroversial statement one can make about the radiographic evaluation of children with FUTIs to identify clinically significant vesicoureteral reflux is that it demonstrates the need for solid evidence-based medicine. A review of the current literature is overwhelming and often conflicting due to differences in definitions and criteria for outcomes. There is controversy in the timing and methods of diagnosis as well as management. Few well-designed, appropriately powered randomized studies have been published on this subject. Thus, we have traditionally employed a homogenous overtreatment strategy in order to avoid undertreating patients with FUTIs. The only consistently emerging theme in this debate is the need for individualized therapy rather than a blanket recommendation regarding the workup and treatment for this condition.

## Figures and Tables

**Table 1:** Advantages and Disadvantages of Bottom-Up versus Top-Down Methodology.

	Advantages	Disadvantages	Comments
*Bottom-Up* *VCUG*	Widely available with reproducible techniques and interpretation Identifies lower urinary tract correctable anomalies	Requires catheterization Does not identify all vulnerable kidneys Exposure to focused ionizing radiation May overtreat cases that are not clinically significant	VUR will predispose kidneys to pyelonephritis and scarring but the relationship is not 1 : 1 Many cases of VUR spontaneously resolve

*RUS*	Noninvasive No ionizing radiation Widely available	Fails to alter management as many abnormalities are now detected in utero Not a functional study	Gross anatomic assessment to complement VCUG

*Top-Down* *DMSA*	Identifies kidneys vulnerable to injury Avoids VCUG (and catheterization) in a subset of patients Acute phase scans have high sensitivity and specificity for clinically significant VUR and recurrent UTI	Heterogeneity in availability, quality, and interpretation Requires intravenous access and long appointment times Requires sedation in young children Misses cases of VUR If only a late-phase scan is performed, it may miss patients at risk for recurrent FUTI Exposure to diffuse ionizing radiation	Early scans may show parenchymal inflammation but only 40% may progress to scarring If only late phase scans are performed, cases of repeat FUTI will be missed
